# Adjuvant high-dose medroxyprogesterone acetate for early breast cancer: 13 years update in a multicentre randomized trial

**DOI:** 10.1054/bjoc.2001.1829

**Published:** 2001-07

**Authors:** C Focan, M Beauduin, E Salamon, J de Greve, G de Wasch, J P Lobelle, F Majois, A Tagnon, J Tytgat, S van Belle, R Vandervellen, A Vindevoghel, for the Adjuvant Breast Cancer Project Belgium

**Affiliations:** Saint-Joseph Clinics-Liège; Jolimont Hospital – Haine Saint-Paul; Sainte-Elisabeth Clinic – Namur; Oncology Centre, AZ-VUB; Jette; Pharmacia & Upjohn, Benelux – Brussels; Institut Medico-Chirurgical, Tournai; Heilige Hart Clinic, Roeselaere; Oncology Centre University – Gent; Saint-Michel Clinic – Brussels, Belgium

## Abstract

The authors updated their report on a randomized trial initiated in 1982 comparing, in early breast cancer, high-dose IM Medroxyprogesterone acetate (HD-MPA) adjuvant hormonotherapy during 6 months with no hormonotherapy; node-positive patients also received 6 courses of IV CMF (day 1, day 8; q.4 weeks). 246 node-negative (NN) and 270 node-positive (NP) patients had been followed for a median duration of 13 years. Previous results were confirmed in this analysis on mature data. In NN patients, relapse-free survival (RFS) was improved in the adjuvant hormonotherapy arm, regardless of age while overall survival (OAS) was also increased in younger (less then 50 years) patients. In the whole group of NP patients, no difference was seen regarding RFS or OAS. However, an age-dependant opposite effect was observed: younger patients (< 50) experienced a worse and significant outcome of relapse-free and overall survivals when receiving adjuvant HD-MPA while older patients (> = 50) enjoyed a significant improvement of their relapse-free survival. For both NN and NP patients, differences in overall survivals observed in older women with a shorter follow-up, were no longer detected. © 2001 Cancer Research Campaign http://www.bjcancer.com


British Journal of Cancer (2001) 85(1), 1–8
© 2001 Cancer Research Campaign
doi: 10.1054/ bjoc.2001.1829, available online at http://www.idealibrary.com on IDEAL®

http://www.bjcancer.com

### Adjuvant high-dose medroxyprogesterone acetate for early breast cancer: 13 years update in a multicentre randomized trial

**C Focan[1], M Beauduin[2], E Salamon[3], J de Greve[4], G de Wasch[4], JP Lobelle[5], F Majois[2], A Tagnon[6], J Tytgat[7], S van Belle[8], R Vandervellen[9] and A Vindevoghel[3] for the Adjuvant Breast Cancer Project Belgium**

[1]Saint-Joseph Clinics-Liège; [2]Jolimont Hospital – Haine Saint-Paul; [3]Sainte-Elisabeth Clinic – Namur; [4]Oncology Centre, AZ-VUB; Jette; [5]Pharmacia & Upjohn, Benelux – Brussels; [6]Institut Medico-Chirurgical, Tournai; [7]Heilige Hart Clinic, Roeselaere; [8]Oncology Centre University – Gent; [9]Saint-Michel Clinic – Brussels, Belgium

**Summary** The authors updated their report on a randomized trial initiated in 1982 comparing, in early breast cancer, high-dose IM Medroxyprogesterone acetate (HD-MPA) adjuvant hormonotherapy during 6 months with no hormonotherapy; node-positive patients also received 6 courses of IV CMF (day 1, day 8; q.4 weeks). 246 node-negative (NN) and 270 node-positive (NP) patients had been followed for a median duration of 13 years. Previous results were confirmed in this analysis on mature data. In NN patients, relapse-free survival (RFS) was improved in the adjuvant hormonotherapy arm, regardless of age while overall survival (OAS) was also increased in younger (less then 50 years) patients. In the whole group of NP patients, no difference was seen regarding RFS or OAS. However, an age-dependant opposite effect was observed: younger patients (< 50) experienced a worse and significant outcome of relapse-free and overall survivals when receiving adjuvant HD-MPA while older patients (> = 50) enjoyed a significant improvement of their relapse-free survival. For both NN and NP patients, differences in overall survivals observed in older women with a shorter follow-up, were no longer detected. © 2001 Cancer Research Campaign http://www.bjcancer.com

Meta-analyses and overviews performed by the Early Breast Cancer Trialists' Collaborative Group (EBCTCG) emphasized the pivotal role of tamoxifen as adjuvant hormonal treatment in early breast cancer patients with positive oestrogen receptors (ER) (Fisher et al, 1997; EBCTCG, 1998). This effect was observed regardless of age and tended to increase with treatment duration (greater effect with longer treatment) and with time as both the improvement in survival and the reduction of controlateral breast cancer went on throughout the first 10 years (EBCTCG, 1998). However, the incidence of endometrial cancers also increased with the duration of tamoxifen treatment (EBCTCG, 1998). The same EBCTC group also demonstrated the value of adjuvant oophorectomy in premenopausal women suffering from a primary breast cancer (EBCTCG, 1996); further trials showed that the adjuvant effect of oophorectomy was restricted to ER-positive tumours (Stewart and Everington, 1995).

In metastatic breast cancer, tamoxifen has been the preferred primary endocrine treatment, particularly for post-menopausal women, for many years (Rubens, 1993). Comparative evaluations of the relative efficacy of tamoxifen and progestogens (especially at high dosages) provided evidence of an at least equipotent or even better antitumoural activity of the progestogens (Mattson, 1980; Löber et al, 1981; Van Veelen et al, 1986; Muss et al, 1988; Muss et al, 1990; Castiglione-Gertsch et al, 1993; Gill et al, 1993). Moreover, after having crossed-over, progestogens retained some antitumour efficacy after tamoxifen's failure (Mattson, 1980; Löber et al, 1981; Van Veelen et al, 1986; Muss et al, 1988; Muss et al, 1990; Castiglione-Gertsch et al, 1993; Gill et al, 1993). Some trials (Castiglione-Gertsch et al, 1993) suggested that tamoxifen's primacy may be challenged particularly by the use of progestogens in patients suffering from bone metastases (Muss et al, 1988; Muss et al, 1990; Castiglione-Gertsch et al, 1993; Gill et al, 1993). In those trials, as already observed, the dose of progestogen (Mattson, 1980; Löber et al, 1981; Cavalli et al, 1984; Tchekmedyian et al, 1986; Van-Veelen et al, 1986) could be of paramount for determining the antitumor efficacy of these agents: high doses of drugs could significantly increase the response rate and even, in some observations, prolong time to treatment failure and survival (Mattson, 1980; Löber et al, 1981; Cavalli et al, 1984; Tchekmedyian et al, 1986; Van Veelen et al, 1986). Furthermore, like first-line hormonotherapy for advanced ER positive breast cancers, in premenopausal patients, HD-MPA developed antitumour activity at least equivalent, if not superior, to oophorectomy (Martoni et al, 1991). The addition of medroxyprogesterone acetate in high dosage (i.e. at least 500 mg day$^{-1}$) to combined chemotherapy also enhanced initial tumour shrinkage and perhaps prolonged survival in advanced breast carcinoma (Robustelli Della Cuna et al, 1980; Löber et al, 1981).

Although the action of progestins, such as high-dose medroxyprogesterone acetate (HD-MPA), can be explained by interacting

*Received 23 February 2000*
*Revised 2 October 2000*
*Accepted 9 February 2001*

*Correspondence to:* C Focan

Presented at the 6th International Consensus Conference on adjuvant therapy of primary breast cancer (St-Gall-Swiss – February 1998).

with oestrogens, either by inhibition of gonadotrophin secretion or down-regulation of the oestrogen receptors, they may also have a direct inhibitory effect on tumour cell growth after binding to progesterone receptors (Löber et al, 1981; Blossey et al, 1984; Paridaens et al, 1986; Rubens, 1993; Hyder et al, 1998). HD-MPA induces a significant blockage of the hypothalamo-hypophyso-gonadal and adrenal axes (Blossey et al, 1984; van Veeler et al, 1985). Therefore, in premenopausal women, HD-MPA is able to provide both castration and adrenal suppression (Löber et al, 1981; Blossey et al, 1984; Van Veelen et al, 1985; Paridaens et al, 1986; Rubens, 1993; Hyder et al, 1998).

On those bases, in 1982, the Adjuvant Breast Cancer Project Belgium initiated a multicentric randomized trial aiming at evaluating the impact of adjuvant therapy with HD-MPA intramuscularly administered in women submitted to surgery for early breast cancer (Focan et al, 1986; Focan et al, 1989, 1990, 1995, 1996). The treatment had been administered for 6 months in half of the patients; node-positive patients received also CMF (IV; days 1 and 8) during 6 months (Focan et al, 1986, 1990). We reported previously on tolerance of treatments: haematological and general tolerances to CMF were improved in the HD-MPA group; classical side-effects of MPA (i.e., perspiration, tremor, cramps, fluid retention or spotting) were recorded in 11–17% of patients who suffered an average weight gain of 7.4 KGs vs 1.2 KGs under controls at the same time (Focan et al, 1986; Wils, 1988; Focan et al, 1989, 1990, 1995, 1996). Women still menstruating usually developed transient amenorrhoea under MPA treatment; amenorrhoea under CMF therapy was, on the other hand, almost universal over 40 years old (Focan et al, 1990, 1995); however detailed follows-up of those phenomena were not mandatory in the trial. Moreover, partly due to a myeloprotective effect of HD-MPA (Wils, 1988; Focan et al, 1990, 1995), the patients receiving this treatment experienced less delay and/or dose reductions of chemotherapy regardless of age and could receive overall higher dose intensities and dose-intensity products of CMF therapy. Younger patients (<50 years) were also able to support higher doses of CMF than older patients; this was observed both for dose intensities and dose-intensity products (Focan et al, 1986; Hryniuk and Levine, 1986; Ng et al, 1989; Focan et al, 1990, 1995). In fact, patients under MPA adjuvant treatment could receive about 90% of the total chemotherapy foreseen while patients under CMF alone received about 70% of the scheduled treatment (Focan et al, 1990, 1995).

In this report, we present the results regarding the outcome of patients (relapse-free and overall survivals) after a 13-year median follow-up. Previous reports were published in full papers in books of proceedings of meetings (Focan et al, 1990, 1995) or as congress abstracts (Focan et al, 1996); the results at 3 years in node-negative patients were the only developed in a peer-reviewed journal (Focan et al, 1989).

### MATERIAL AND METHODS

Detailed methodology was published previously (Focan et al, 1990, 1995). Briefly, patients under 70, with primary stage I or II breast cancer and a Karnofsky index >60 were eligible for the trial; axillary lymph node status (node-negative (NN) or node-positive (NP)) had to be assessed on at least 5 nodes. Hormonal receptors, when available, were assessed by the dextran-coated charcoal assay. After either mastectomy or limited surgery, patients were randomized to receive HD-MPA or no adjuvant treatment. The HD-MPA treatment included an induction period (28 consecutive daily i.m. injections of 500 mg MPA or 5 daily i.m. injections of 500 mg MPA weekly during 5 weeks) followed by a maintenance treatment (500 mg i.m. twice per week during 5 months). Axillary NP patients were also given CMF chemotherapy (cyclophosphamide 500 mg/m$^2$ i.v., methotrexate 40 mg/m$^2$ i.v. and 5-fluorouracil 600 mg/m$^2$ i.v., days 1 and 8, to be repeated every 4 weeks during 6 cycles). Actuarial survivals (relapse-free and overall) were evaluated according to the Kaplan–Meier method while applying Log-rank and Wilcoxon statistical tests (Kaplan and Meier, 1958). Any first breast cancer relapse (local and/or distant) was considered as an event for the RFS; every death regardless of the cause was considered for the OAS. Finally, a Cox's proportional hazards regression analysis was conducted to explain relapse-free and overall survivals as functions of independent variables (Cox, 1972).

### RESULTS

541 patients were randomized between 1982 and 1989: 260 patients in the NN group and 281 patients in the NP group among whom respectively 246 and 270 were fully assessable. The reasons for ineligibility or invalidity have been described elsewhere (Focan et al, 1989, 1995, 1996). The characteristics of both groups are recalled in Table 1. Every patients' characteristic was well balanced but one: a higher number of patients with 1–3 positive axillary nodes and a lower number of patients with more than 4 involved nodes were included by chance within the CMF-alone arm (Table 3). Patients had been followed up for a median time of 13 years.

The relapse-free survival was significantly improved in the NN group treated with HD-MPA (Table 3, Figure 1). After 13 years, RFS was respectively 69% in the MPA group vs 54% in the control group. This difference was confirmed regardless of age (< 50 years: + 22%; ⩾ 50 years: + 10%) and of any other prognostic factor (such as T stage, receptors levels, menopausal status) (Focan et al, 1989, 1990, 1995, 1996). However, this difference could not be clearly translated into an improvement of overall survival despite a significant difference after 5 years in each age subgroup (Focan et al, 1990) and a border line significant effect in favour of the adjuvant hormone arm in patients under 50 years (+ 16%) which was still detectable with more prolonged follow-up ($P$ = 0.06) (Table 3).

Contrary to what was observed in NN patients, no difference was noted in relapse or death rates for the whole group of NP patients. However, when patients were split according to their ages (<50; ⩾ 50 years), clear-cut differences appeared (Table 3). Indeed, patients under 50 years had a worse evolution when treated with the association of CMF and MPA; in this group, not only relapse-free (+30%) but overall survivals as well (+23%) were quite significantly bettered in the group treated with CMF alone. On the contrary, patients over 50 years who were treated with the combined modality had a significantly improved relapse-free interval (+25%) than those treated with CMF alone (Table 3; Figure 2); overall survival of these patients remained marginally improved (+9%). In NP patients as well, other prognostic determinants (T stage, number of positive nodes, hormone receptor levels) did not influence the results described above (Focan et al, 1989, 1990, 1995, 1996). In peculiar, no difference in RFS or OAS could be assessed when patients were analysed according to the negativity (< 10 fmol mg$^{-1}$ prot) or positivity (≥ 10 fmol mg$^{-1}$ prot) of

© 2001 Cancer Research Campaign

**Table 1** Characteristics of patients

| | Node negative | | | Node positive | | |
|---|---|---|---|---|---|---|
| | **Control** | **HD-MPA** | ***P*** | **CMF** | **CMF + HD-MPA** | ***P*** |
| All evaluable patients | 123 | 123 | | 138 | 132 | |
| Premenopausal | 41 | 44 | | 57 | 58 | |
| Perimenopausal | 10 | 18 | | 18 | 10 | |
| Postmenopausal | 66 | 52 | NS | 54 | 54 | NS |
| Unknown | 6 | 9 | | 9 | 10 | |
| Age, years | | | | | | |
| < 50 | 44 | 47 | | 52 | 52 | |
| ≥ 50 | 79 | 76 | NS | 86 | 80 | NS |
| Unknown | 3 | 0 | | 0 | 0 | |
| T1 | 48 | 51 | | 23 | 24 | |
| T2 | 66 | 61 | | 87 | 83 | |
| T3 | 5 | 5 | NS | 19 | 21 | NS |
| Tx | 4 | 6 | | 9 | 4 | |
| ER, fmol $mg^{-1}$ prot | | | | | | |
| < 10 | 28 | 26 | | 41 | 27 | |
| ≥ 10 | 51 | 43 | NS | 65 | 61 | NS |
| Unknown | 44 | 54 | | 32 | 44 | |
| PgR, fmol $mg^{-1}$ prot | | | | | | |
| < 10 | 31 | 29 | | 35 | 23 | |
| > = 10 | 46 | 40 | NS | 70 | 64 | NS |
| Unknown | 46 | 54 | | 33 | 45 | |
| Mastectomy | 73 | 76 | | 114 | 115 | |
| Limited surgery | 49 | 43 | NS | 19 | 16 | NS |
| Radiotherapy | | | | | | |
| Yes | 104 | 85 | | 109 | 109 | |
| No | 17 | 35 | < 0.05 | 26 | 23 | NS |
| Number of positive nodes | | | | | | |
| 1–3 | – | – | | 81 | 60 | |
| 4–9 | – | – | | 34 | 52 | < 0.05 |
| > = 10 | – | – | | 19 | 19 | |
| Unknown | | | | 4 | 1 | |

NS = not significant.

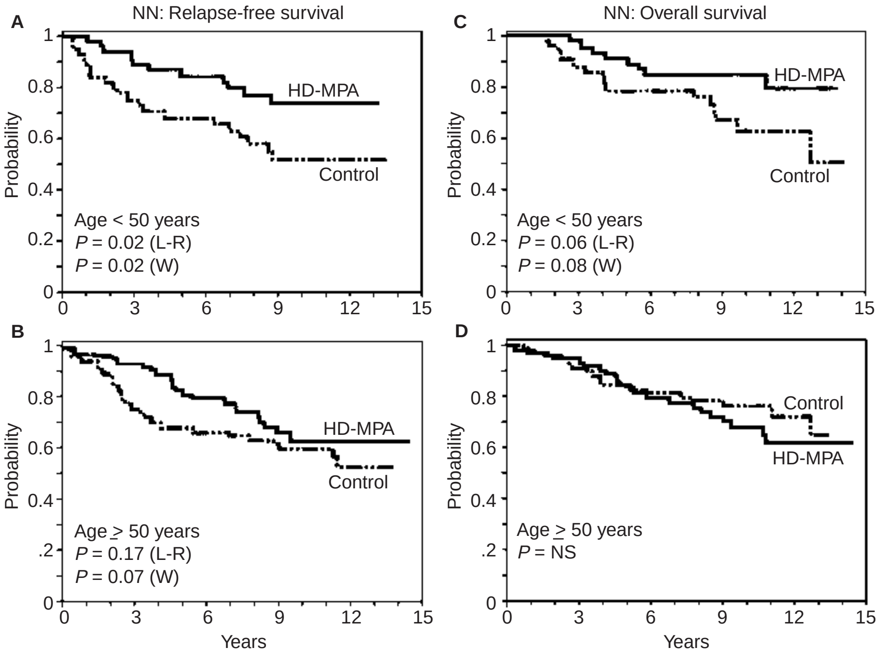

**Figure 1** Actuarial relapse-free and overall survival in node-negative (NN) patients according to age (< 50: A, C; ≥ 50: B, D). In abscissa, the probability of survival. In ordinate, the time in years (L-R: log-rank test; W: Wilcoxon test)

© 2001 Cancer Research Campaign

**Table 2** Distribution of node-positive patients according to number of involved nodes and age

| | CMF | CMF + HD-MPA | *P* |
|---|---|---|---|
| Age < 50 | | | |
| N 1–3 | 33 | 23 | |
| N 4–9 | 11 | 23 | 0.026 |
| N ⩾ 10 | 8 | 5 | |
| Unknown | 0 | 1 | |
| Age ⩾ 50 | | | |
| N 1–3 | 48 | 37 | |
| N 4–9 | 23 | 29 | 0.115 |
| N ⩾ 10 | 11 | 14 | |
| Unknown | 4 | 0 | |

the tumoral receptors (Focan et al, 1989, 1990, 1995, 1996). However receptors levels were only assessed in 60–70% of cases; then, the number of patients in some sub-groups might have been too low to draw any statistical evaluation (see Table 1). Furthermore, results both in younger and older patients were not influenced by the number of positive nodes (Table 4); this observation assumed a special interest for node positive < 50-year-old women, in whom a mean lower number of positive nodes was expressed by chance in the CMF alone arm (Table 1). This could have favoured the final outcome of those patients. However, older patients presented a better evolution with the combined treatment whatever the number of positive nodes (Table 4).

Univariate analyses by the Kaplan–Meier estimates performed on the whole group evidenced a significant influence of, on the

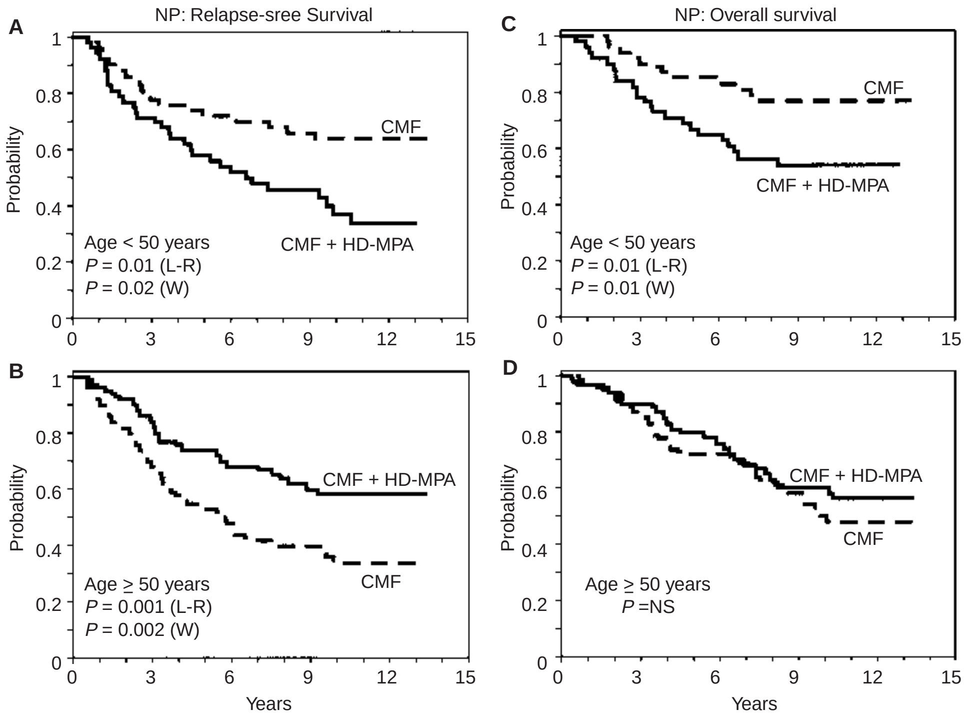

**Figure 2** Actuarial relapse-free and overall survival in node-positive (NP) patients according to age (< 50 : A, C; ≥ 50: B, D). In abscissa, the probability of survival. In ordinate, the time in years (L-R: log-rank test; W: Wilcoxon test)

**Table 3** Actuarial survival (probability ± standard error at 13 years)

| | Node negative | | | | Node positive | | | |
|---|---|---|---|---|---|---|---|---|
| | Control | HD-MPA | *P* | | CMF | CMF + HD-MPA | *P* | |
| | (123) | (123) | w | lg | (138) | (132) | w | lg |
| Relapse-free survival | | | | | | | | |
| Whole group | 0.54 ± 0.05 | 0.69 ± 0.05 | 0.005 | 0.01 | 0.46 ± 0.04 | 0.48 ± 0.05 | NS | NS |
| < 50 years | 0.52 ± 0.08 | 0.74 ± 0.07 | 0.02 | 0.03 | 0.64 ± 0.07 | 0.34 ± 0.07 | 0.03 | 0.01 |
| ⩾ 50 years | 0.55 ± 0.07 | 0.65 ± 0.06 | 0.06 | 0.17 | 0.34 ± 0.06 | 0.59 ± 0.06 | 0.002 | 0.002 |
| Overall survival | | | | | | | | |
| Whole group | 0.70 ± 0.05 | 0.70 ± 0.05 | NS | NS | 0.59 ± 0.05 | 0.56 ± 0.04 | NS | NS |
| < 50 years | 0.65 ± 0.08 | 0.81 ± 0.06 | 0.08 | 0.06 | 0.77 ± 0.06 | 0.54 ± 0.07 | 0.01 | 0.01 |
| ⩾ 50 years | 0.73 ± 0.06 | 0.63 ± 0.06 | NS | NS | 0.47 ± 0.06 | 0.56 ± 0.06 | NS | NS |

W = Wilcoxon test; lg = logrank test.

 *© 2001 Cancer Research Campaign*

**Table 4** Actuarial survival at 13 years according to age and number of positive nodes (probability ± standard error at 13 years)

| | CMF | | CMF + HD-MPA | |
|---|---|---|---|---|
| | RFS | OAS | RFS | OAS |
| *Age < 50* | | | | |
| N 1–3 | 0.73 ± 0.08 | 0.80 ± 0.07 | 0.55 ± 0.11 | 0.64 ± 0.10 |
| N 4–9 | 0.55 ± 0.15 | 0.72 ± 0.13 | 0.20 ± 0.09 | 0.43 ± 0.10 |
| N ⩾ 10 | 0.31 ± 0.18 | 0.62 ± 0.17 | 0.40 ± 0.22 | 0.40 ± 0.22 |
| N ⩾ 4 | 0.44 ± 0.12 | 0.68 ± 0.11 | 0.18 ± 0.08 | 0.42 ± 0.09 |
| *Age* ⩾ 50 | | | | |
| N 1–3 | 0.38 ± 0.07 | 0.57 ± 0.08 | 0.72 ± 0.08 | 0.68 ± 0.08 |
| N 4–9 | 0.32 ± 0.12 | 0.41 ± 0.13 | 0.47 ± 0.10 | 0.56 ± 0.10 |
| N ⩾ 10 | 0.18 ± 0.12 | 0.33 ± 0.15 | 0.35 ± 0.17 | 0.63 ± 0.13 |
| N ⩾ 4 | 0.28 ± 0.09 | 0.41 ± 0.10 | 0.45 ± 0.08 | 0.57 ± 0.08 |

All $P \geq 0.10$ except for:
RFS-Age ≥ 50 N1–3: $P = 0.038$ (W); 0.014 (lg); N ≥ 10: $P = 0.027$ (W); 0.056 (lg); N ≥ 4: $P = 0.008$ (W); 0.025 (lg); OAS-Age < 50 N1–3: $P = 0.077$ (W); N ≥ 4: $P = 0.090$ (W)

**Table 5** Multivariate analysis – Cox model

| | Node negative | | | | Node positive | | | |
|---|---|---|---|---|---|---|---|---|
| | RFS | | OAS | | RFS | | OAS | |
| | Variables | *P* | Variables | *P* | Variables | *P* | Variables | *P* |
| Whole Group | Treatment | 0.14 | ER-cat | 0.14 | N Pos-cat | 0.0097 | N Pos-cat | 0.003 |
| < 50 years | Treatment | 0.006 | | NS | N Pos-cat | 0.002 | N Pos-cat | 0.002 |
| | ER-cat | 0.08 | | | Treatment | 0.09 | Treatment | 0.04 |
| | Age | 0.04 | | | PGR-cat | 0.12 | | |
| ≥ 50 years | ER-cat | 0.015 | Age | 0.11 | Treatment | 0.01 | | NS |
| | Treatment | 0.04 | | | | | | |
| | Age | 0.07 | | | | | | |

Notes: Variables with $P \geq 0.15$ were rejected by the model (quoted as NS not statistically significant). ER (PGR)-cat-oestrogen (progestogen) receptor category (negative; positive ≥ 10 fmol mg$^{-1}$ prot; unknown not included). N Pos-cat = category of positive nodes (0–3; 4–10; ≥ 10)

one side, the T stage on relapse-free survival ($P < 0.02$) in NN patients and, on the other side, the number of positive axillary lymphnodes (1–3; 4–9; ≥10) on relapse-free ($P = 0.005$) and overall ($P = 0.01$) survival in NP patients. In multivariate evaluations (Table 5), treatment and ER-category (negative < 10 or positive ≥ 10 fmol mg$^{-1}$ protein) were independent variables in NN patients. In node-positive patients, the number of positive nodes, as categorized above, and the treatment were also pointed out especially in under 50-year-old women.

### DISCUSSION

In the present study, HD-MPA was administered intramuscularly. When the study started in 1982, it was not known yet whether i.m. and oral routes would have a similar antitumor effect for advanced breast cancer. However, identical antitumor effects for each way of administration were shown in 2 subsequent randomized trials in advanced disease (Paridaens et al, 1986; Beex et al, 1987). Regarding pharmacokinetics, the i.m. route was thought to prolong the exposure time of tumour cells (Blossey et al, 1984; Canney et al, 1988; Etienne et al, 1992). In an adjuvant situation, HD-MPA intramuscularly given during 6 months would in fact accomplish exposure to the drug for at least 3 months more (Blossey et al, 1984; Canney et al, 1988; Etienne et al, 1999). Regarding the chosen dose, it was shown in advanced disease that no further tumour regression could be obtained from daily MPA IM doses over 500 mg during the loading phase (Robustelli Della Cuna et al, 1980; Tamassia et al, 1982). Moreover, in a randomized evaluation, HD-MPA had developed a better antitumoral effect than that with low dosages of the drug (Cavalli et al, 1984). Those findings support our choice for both dose level and route of administration.

From a general point of view, we estimate that we have no data strong enough to develop definitive considerations on the results regarding the level of receptors in tumours. In fact, the trial was initiated in 1982, at a time when the determination of tumour receptors was not routinely performed in the oncological practice and when the dextran-coated charcoal assay was the only method available in a few centres to which tumour samples of our study were referred. Therefore, these assessments were not obtainable for all tumours, especially smaller ones; so, hormone receptors determination was lacking in 30–40% of our cases (Table 1). Then, despite a sufficiently high number of patients to conclude at group levels, we remain cautious regarding the results provided for

*© 2001 Cancer Research Campaign*

sub-groups, especially the premenopausal ones. In spite of these restrictions, the tumour receptor level emerged as an independent prognostic variable through the multivariate analysis (Table 5).

Since we were specially interested to try to correlate some of our results (especially in < 50 years patients) with the tumour receptors levels, we have considered to reassess ER status by immunohistochemical methods now routinely applied (Andersen, 1992; Pertschuk et al, 1994; Alberts et al, 1996). Every patient in the study was analysed between 1981 and 1987 at a time when ER status could not be routinely determined by immunohistochemical techniques. Every retrospective assessment would be besmirched by practical and methodological difficulties. Indeed, we would be unable to correlate actual data with immunochemistry with previous ones obtained with the dextran-coated charcoal assays. Therefore, from a methodological point of view, survival analyses considering immunohistochemical data would have to be reassessed as separate subsets. Moreover, patients who were entered in the trial were coming from many different hospitals outside reference hospitals, in which they were treated. This would seriously complicate the searches to obtain blank slides at the present time. The pathologists are obliged by Belgian law to retain paraffin-embedded sections of human samples for at least 15 years; this means that for patients included before 1985, we must expect to discover that many samples are no further available. From a pathological point of view, a considerable loss of immunoreactivity has been observed in paraffin-embedded specimens fixed in formaline or Bouin in comparison to sections from frozen tissues (Andersen, 1992; Pertshuk et al, 1994). Moreover, the majority of results shows discordances between dextran-coated charcoal assay and immunohistochemical determinations in the measurements of ER status in premenopausal females (Alberts et al, 1996). This was precisely the group of patients for whom we would have been interested to obtain more detailed information on the receptors status due to the observed survival results. Finally, in multivariate analysis, only ER status determined by dextran-coated assay performed better than ER status assessed by immunohistochemistry as an independent prognostic variable regarding disease-free and overall survival (Andersen, 1992; Alberts et al, 1996). Due to all these practical and methodological difficulties and to the final split of patients in various prognostic subcategories (basically regarding age), we considered that we would ultimately not have interest in, a posteriori, reassessing the tumour receptors levels by immunohistochemical methods.

With a 13-year-median follow-up, we confirm previous reports on the adjuvant activity of HD-MPA in NN early breast cancer (Focan et al, 1989; 1990, 1995) with a better RFS, regardless of age. Concerning survival, after a longer-term observation, only women under 50 years seemed to benefit the most from the treatment, since the early observed adjuvant effect tended to disappear in older subjects. Precisely in premenopausal patients, according to the multivariate assessment, the adjuvant impact of MPA seems to be stronger (Focan et al, 1989, 1990, 1995; EBCTCG, 1998) in patients with receptor-positive tumours (Table 5).

These results must be considered in the light of those obtained in premenopausal (or ≤ 50 years) women with adjuvant ovarian ablation or tamoxifen use (EBCTCG, 1996; Fisher et al, 1997; EBCTCG, 1998). The EBCTC group recently confirmed, in a meta-analysis on more than 2000 women, the significant adjuvant activity of an ovarian ablation; RFS and OAS were significantly improved in the castrated group independently of the nodal status (EBCTCG, 1996). This adjuvant effect seems to be limited to receptor-positive tumours (Stewart and Everington, 1995). In those patients, the magnitude of the benefit may equal that obtained from an adjuvant chemotherapy (Stewart and Everington, 1995; Ejlertsen et al, 1999). The adjuvant effect of medical castration induced by LH-RH analogues, such as goserelin, may also be potentiated by the concomitant use of tamoxifen (Rutqvist, 1999); this association may even outshine the effect of CMF (Jakesz et al, 1999). At this point, it must be remembered that part of the HD-MPA hormonal activity could also be related to a general hypophyseal adrenal and gonadal blockage; so, part of its antitumoral activity could be related to this castrative effect (Van Veelen et al, 1985; Martoni et al, 1991; Paridaens et al, 1986).

In ER-positive breast cancer patients, regardless of age, the EBCTC group also confirmed a quite significant adjuvant impact with an improvement of survival and a decrease of contralateral breast cancer from the use of tamoxifen (EBCTCG, 1998). Of interest, as reported for tamoxifen, we observed a sustained adjuvant effect for both relapse-free and overall survival in younger NN patients. In NN patients over 50 years, the significant decrease in relapse-free survival observed after 5 years (Focan et al, 1990, 1996) tended to diminish and finally was not detectable any more after 9 years. This transient effect was not translated into an overall survival benefit and was in opposition with the observation that in tamoxifen-treated patients the outcome improved with time (EBCTCG, 1998). However our therapeutic programme was limited to 6 months. This temporary exposition to MPA could have been too short; for tamoxifen indeed, it was previously demonstrated clearly that the longer the treatment the better the results in terms of relapse or survival (5 years > 2 years > 1 year) (EBCTCG, 1998).

In NP patients, we were puzzled with the results which were clearly influenced by the age and/or the menopausal status of the host. As those patients received also CMF chemotherapy, the impact of the latter on the menstrual status of premenopausal women as well as the biology and kinetics of the breast tumour has to be questioned. Since the doses of chemotherapy were higher in patients receiving MPA (Focan et al, 1995, 1996), especially in younger ones (Focan et al, 1986, 1995, 1996), the hypothesis of a dose–response relationship is not sustainable. In women under 50 years, the prognosis was better in the group under CMF only, which had also received the lowest doses of chemotherapy. Therefore, hormone manipulation with HD-MPA seemed to prevent the adjuvant activity of chemotherapy in premenopausal patients although higher dose levels of chemotherapy could be safely delivered. Could MPA block cells outside the cell cycle (in G0–G1 phases) and prevent then cytocidal activity of chemotherapy? Could the general hypophyseal and adrenal axes blockages prevent the adjuvant effect of chemotherapy in younger hosts? Could the hormonal milieu influence outcome under combined treatment? We must recall here that the general use of tamoxifen in premenopausal breast cancer patients still remains a matter of discussion (Andersson et al, 1999; Bramwell and Pritchard, 1999; Hutchins et al 1999). In some groups of patients, its administration may be harmful: this has been observed by several authors precisely when a chemotherapy was administered concomitantly (Hutchins et al, 1999); this observation was obvious for receptor-negative tumours (Andersson et al, 1999; Hutchins et al, 1999). In our trial, in postmenopausal women or in women over 50 years the combined modality approach improved the results achievable with the only chemotherapy. This observation coincides with previous reports suggesting the potentiation between chemo- and hormonotherapy

© 2001 Cancer Research Campaign especially in patients with hormone receptor-containing tumours at early or advanced stages of the disease (Rubens, 1993).

An unexpected imbalance in the number of positive nodes between both arms of treatment was observed (Table 1 and 2). Since the number of positive nodes was lower in the CMF arm, especially in younger patients, we had to exclude a possible interaction between treatment and nodal status. In fact, this hypothesis was not retainable:

(1) because the imbalance regarding the number of involved nodes was expressed partly in premenopausal, but also in post-menopausal woman in whom the combined treatment did better than CMF alone (Table 4);
(2) because both in pre-and post-menopausal patients, exactly the same trend in survivals (better for CMF alone in younger and better for CMF + MPA in older subjects) was obvious, if the impact of treatment was reanalysed after splitting subjects according to the number of nodes (1–3; 4–9; ⩾ 10 or ⩾ 4) (Table 4). Of course, due to the limited number of patients in constructed sub-groups, some differences which were statistically significant when the whole group was considered were no longer assessable, but they remain of the same relative amplitudes as that of the whole group (Table 4).

Several other trials assessed the value of adjuvant HD-MPA in NP early breast cancer (Pannuti et al, 1988; Hupperets et al, 1993). Observations similar to ours were obtained in NP patients treated in 2 other trials with HD-MPA using a schedule comparable to ours with a 6-month programme and CMF or FAC (5-fluorouracil, adriamycin and cyclophosphamide) chemotherapy (Pannuti et al, 1988; Hupperets et al, 1993). In those trials, as well, only older patients seemed to better benefit from the combined treatment modality (Pannuti et al, 1988; Hupperets et al 1993; Focan et al, 1995).

### CONCLUSION

Adjuvant HD-MPA therapy increased the relapse-free survival (and overall survival in patients under 50 years) in NN early breast carcinoma. In NP patients who also received CMF therapy, a positive effect was proved in patients over 50 years; in younger patients (<50 years), the addition of HD-MPA to CMF chemotherapy seemed detrimental.

Based on our results, HD-MPA may be considered as an alternative hormonal adjuvant treatment instead of ovariectomy or tamoxifen in the case of premenopausal or <50-year-old women, preferably with ER-positive node-negative breast cancers. HD-MPA may be proposed only with caution to early node negative ⩾ 50-year-old patients. In younger women with node-positive cancer, candidates to chemotherapy, it may not be administered at the same time as chemotherapy. The lack of global survival improvement in patients over 50 despite a clear effect on relapse-free survival may suggest that the duration of HD-MPA treatment (6 months) had been too short. Therefore, since HD-MPA has a scientific effect only on some patient's categories and since the clinical toxicity (i.e., an important weight gain) may be significant (Focan et al, 1986, 1989, 1990, 1995), its routine use may not be recommended without direct evaluation in relation to tamoxifen.

### ACKNOWLEDGEMENTS

This work was supported by a grant from Farmitalia Carlo Erba Benelux. We gratefully thank Drs U Bunescu, N Dehasque, P Driesschaert, D Focan-Henrard, E Longeval, H Spapen, B Vanderlinden, P Vanderhoven and A Warnier for their participation in this trial.R Franssen and M Focan are also gratefully acknowledged respectively for excellent editorial assistance and for reviewing the English formulation.

### REFEREN1CES

Alberts SR, Ingle JN, Roche PR, Cha S, Wold LE, Favr GH Jr, Knook JE and Wieand HS (1996) Comparison of estrogen receptor determination by a biochemical ligand binding assay and immunohistochemical staining with monoclonal antibody ER1D5 in females with lymph node positive breast carcinoma entered on prospective clinical trials. *Cancer* **78**(4): 764–772

Andersen J (1992) Determination of estrogen receptors in paraffin-embedded tissue. Technic and the value in breast cancer treatment. *Acta Oncol* **31**(6): 611–627

Andersson M, Kamby C, Jensen MB, Mouridsen H, Ejlerstsen B, Dombernowsky P, Rose C, Cold S, Overgaard M, Andersen J and Kjaer M (1999) Tamoxifen in high-risk premenopausal women with primary breast cancer receiving adjuvant chemotherapy. Report from the Danish Breast Cancer Co-operative Group DBCG 82B Trial. *Eur J Cancer* **35**(12): 1659–1666

Beex L, Burghouts J, van Turnhout J, Breed W, Hillen H, Holdrinet A, Boetius G, Hoogendoorn G, Doesburg W and Verhulst M (1987) Oral versus i.m. administration of high-dose medroxyprogesterone acetate in pretreated patients with advanced breast cancer. *Cancer Treat Rep* **71**: 1151–1156

Blossey HC, Wander HE, Koebberling J and Nagel GA (1984) Pharmacokinetic and pharmacodynamic basis for the treatment of metastatic breast cancer with high-dose medroxyprogesterone acetate. *Cancer* **54**: 1208–1215

Bramwell VHC and Pritchard KI (1999) Tamoxifen added to adjuvant chemotherapy in premenopausal women with early breast cancer: is it standard practice or still a subject for study? *Eur J Cancer* **35**(12): 1625–1627

Canney PA, Dowsett M and Priestman TJ (1988) The pharmacokinetics of medroxyprogesterone acetate following two different loading dose schedules in advanced carcinoma of the breast. *Br J Cancer* **58**(1): 73–76

Castiglione-Gertsch M, Pampallona S, Varini M, Cavalli F, Brunner K, Senn HJ, Goldhirsch A and Metzger U (1993) Primary endocrine therapy for advanced breast cancer: to start with tamoxifen or with medroxyprogesterone acetate? *Ann Oncol* **4**: 735–740

Cavalli F, Goldhirsch A, Jungi F, Martz G, Mermillod B and Alberto P, (for the Swiss Group for Clinical Cancer Research) (1984) Randomized trial of low-versus high-dose medroxyprogesterone-acetate in the induction treatment of postmenopausal patients with advanced breast cancer. *J Clin Oncol* **2**: 414–419

Cox DR (1972) Regression models and life-tables (with discussion). *J. R. Stat S [B]* **34**: 187–220

Early Breast Cancer Trialists' Collaborative Group (1998) Tamoxifen for early breast cancer: an overview of the randomised trials. *The Lancet* **351**: 1451–1467

Early Breast Cancer Trialists' Collaborative Group (1996) Ovarian ablation in early breast cancer: overview of the randomised trials. *The Lancet* **348**: 1189–1199

Ejlertsen B, Dombernowsky P, Mouridsen HT, Kamby C, Kjaer M, Rose C, Andersen KW, Jensen MB, Bengtsson NO and Bergh J (1999) Comparable effect of ovarian ablation (OA) and CMF chemotherapy in premenopausal hormone receptor positive breast cancer patients (PRP). *Proc of ASCO*, **18**: 660 (abstr. 248)

Etienne MC, Milano G, Frenay M, Renee N, François E, Thyss A, Schneider M and Namer M (1992) Pharmacokinetics and pharmacodynamics of medroxyprogesterone acetate in advanced breast cancer patients. *J Clin Oncol 1992* **10**(7): 1176–1182

Fisher B, Dignam J, Wolmark N, DeCillis A, Emir B, Wickerham DL, Bryant J, Dimitrov NV, Abramson N, Atkins JN, Shibata H, Deschenes L and Margolese RG (1997) Tamoxifen and chemotherapy for lymph node-negative, estrogen receptor-positive breast cancer. *J Natl Cancer Inst* **89**(22): 1673–1682

Focan C, Baudoux A, Beauduin M, Bunescu U, Dehasque N, Dewasch G, Lobelle JP, Longeval E, Majois F, Salamon E, Spapen H, Tagnon A, Tytgat J, Van Belle S, Vanderlinden B, Vandervellen R and Vindevoghel A (1986) Improvement of hematological and general tolerance to CMF by high-dose medroxyprogesterone-acetate (HD-MPA) adjuvant treatment for primary node positive breast cancer (analysis of 100 patients). *Anticancer Res* **6**: 1095–1100

Focan C, Baudoux A, Beauduin M, Bunescu U, Dehasque N, Dewasch G, Lobelle JP, Longeval E, Majois F, Mazy V, Nickers P, Salamon E, Tagnon A, Tytgat J, van Belle S, Vanderlinden B, Vandervellen R and Vindevoghel A (1989) Adjuvant treatment with high dose medroxyprogesterone-acetate in node-

*© 2001 Cancer Research Campaign* 

negative early breast cancer. A 3-year interim report on a randomized trial. *Acta Oncologica* **28**: 237–250
Focan C, Beauduin M, Salamon E, De Wasch G, Driesschaert P, Lobelle JP, Longeval E, Majois F, Tagnon A, Tytgat J, Van Belle S, Vanderlinden B, Vanderhoven P, Vandervellen R, Vindevoghel A and Warnier A (1990) Influence of age, node involvement and CMF chemotherapy on the outcome of early breast cancer treated with high dose medroxyprogesterone acetate (HD-MPA) as adjuvant hormonotherapy: 5 years Results of a randomized trial, in SE Salmon (ed): *Adjuvant Therapy of Cancer VI*., pp 319–329
Focan C, Beauduin M, Salamon E, De Grève J, De Wasch G, Lobelle JP, Majois F, Tagnon A, Tytgat J, van Belle S, Vandervellen R and Vindevoghel A (1995) High-dose medroxyprogesterone acetate as adjuvant hormonotherapy in early breast cancer: 9 years results of a multicenter randomized trial. In Jonat W, Kaufmann M, Munk K (eds): Hormone-Dependent Tumors. Basic Research and Clinical Studies, vol 50, pp 145–158. *Contrib Oncol.* Basel, Karger
Focan C, Beauduin M, Salamon E, De Gréve J, De Wasch G, Lobelle JP, Majois F, Tagnon A, Tytgat J, van Belle S, Vandervellen R and Vindevoghel A (1996) High dose medroxyprogesterone acetate (HD-MPA) as adjuvant hormonotherapy for early breast cancer. Ten years results of a multicenter randomized trial. *Acta Clin Belgica* **51-3**:194
Gill PG, Gebski V, Snyder R, Burns I, Levi J, Byrne M and Coates A (1993) Randomized comparison of the effects of tamoxifen, megestrol acetate, or tamoxifen plus megestrol acetate on treatment of response and survival in patients with metastatic breast cancer. *Ann Oncol* **4**(9): 741–744
Hryniuk WM and Levine MN (1986) Analysis of dose intensity for adjuvant chemotherapy trials in stage II breast cancer. *J Clin Oncol* **4**: 1162–1170
Hupperets PSGJ, Wils J, Volovics L, Schouten L, Fickers M, Bron H, Schouten H, Jager J, de Jong J and Beex L (1993) Adjuvant chemo-hormonal therapy with cyclophosphamide, doxorubicin and 5-fluorouracil (CAF) with or without medroxyprogesterone acetate for node-positive breast cancer patients. Update at 7 year follow-up. *Ann Oncol* **4**: 295–301
Hutchins L, Green S, Ravdin P, Lew D, Martino S, Abeloff M, Lyss A, Henderson C, Alfred C, Dakhil S, Pierce J, Goodwin W, Caton J, Rivkin S, Chapman R and Osborne K (1999) CMF versus CAF +/– Tamoxifen in high-risk node-negative breast cancer patients and a natural history follow-up study in low-risk node negative patients: update of tamoxifen results. *Breast Cancer Research and Treatment* **57**(1): 25
Hyder SM, Murthy L and Stacel GM (1998) Progestin regulation of vascular endothelial growth factor in human breast cancer cells. *Cancer Res* **58**(3): 392–395
Jakesz R, Hausmaninger H, Samonigg H, Kubista E, Depisch D, Fridrik M, Stierer M, Gnant M, Steger G, Kolb R, Jatzko G, Hofbauer F, Reiner G and Luschin-Ebengreuth G (1999) Comparison of adjuvant therapy with tamoxifen and goserelin vs CMF in premenopausal stage I and II hormone-responsive breast cancer patients: Four-year results of Austrian breast cancer study group (ABCSG) trial 5. *Proc of ASCO* **18**: abstr. 250
Kaplan EL and Meier P (1958) Non parametric estimation from incomplete observations. *J Am Stat Assoc* **53**: 457–481
Löber J, Rose C, Salimtschik M and Mouridsen HT (1981) Treatment of advanced breast cancer with progestins. A review. *Acta Obstet Gynecol Scand* (suppl 101) 39–46
Martoni A, Longhi A, Canova N and Pannuti F (1991) High-dose medroxyprogesterone acetate versus oophorectomy as first-line therapy of advanced breast cancer in premenopausal patients. *Oncology* **48**(1): 1–6
Mattsson W (1980) A phase III trial of treatment with tamoxifen versus treatment with high-dose medroxyprogesterone-acetate in advanced post-menopausal breast cancer, in lacobelli S and di Marco A (ed): Role of medroxyprogesterone in endocrine-related tumors – Progress in cancer research and therapy. Vol 15, pp 65–71, New York, Raven Press
Muss HB, Case LD, Capizzi RL, Cooper MR, Cruz J, Jackson D, Richards F, Powell BL, Spurr CL and White D (1990) High-versus standard-dose megestrol acetate in women with advanced breast cancer. A phase III trial of the Piedmont Oncology Association. *J Clin Oncol* **8**: 1797–1805
Muss HB, Wells B, Paschold EH, Black WR, Coopper MR, Capizzi RL, Christian R, Cruz JM, Jackson DV and Powell BL (1988) Megestrol acetate versus tamoxifen in advanced breast cancer: 5 year analysis – a phase III trial of the Piedmont Oncology Association. *J Clin Oncol* **6**: 1098–1106
Ng V, Ragaz J, Spinelli HJ and Jackson S (1989) Introduction of cumulative dose intensity product (DIP) for measuring the delivered dose intensity (DI) of chemotherapy – Analysis of the British Columbia Breast Cancer Randomized Study. *Proceedings of Am Soc Clin Oncol* **8**: 47 (Abstr 181)
Pannuti F, Martoni A, Cilenti G, Camaggi CM and Fruet F (1988) Adjuvant therapy for operable breast cancer with medroxyprogesterone acetate alone in postmenopausal patients or in combination with CMF in premenopausal patients. *Eur J Cancer Clin Oncol* **24**(3): 423–429
Paridaens R, Becquart D, Michel J, Vanderlinden B, Longueville J, Majois F, Beauduin M, Focan C, Wildiers J, Bernheim J, Lobelle JP, Arrigo C, Sylvester RJ and Heuson JC (1986) Oral versus intramuscular high-dose medroxyprogesterone (HD-MPA) in advanced breast cancer. *Anticancer Res* **6**: 1089–1094
Pertschuk LP, Kim YD, Axiotis CA, Braverman AS, Carter AC, Eisenberg KB and Braithwaite LV (1994) Estrogen receptor immunocytochemistry: the promise and the perils. *J Cell Biochem Suppl* **19**: 134–137
Robustelli Della Cuna G, Calciati A, Bernardo Strada MR, Bumma C and Campio L (1980) High-dose medroxyprogesterone-acetate (HD-MPA) combined with chemotherapy for metastatic breast carcinoma, in lacobelli S, Di Marco A (ed): Role of medroxyprogesterone acetate in endocrine-related tumors, pp 53–64. New York, Raven Press
Rubens RD (1993) Towards improved endocrine therapy for advanced breast cancer. *Ann Oncol* **4**: 712–713
Rutqvist LE (1999) Zoladex and tamoxifen as adjuvant therapy in premenopausal breast cancer: a randomised trial by the cancer research campaign (C.R.C.) Breast Cancer Trials Group, the Stockholm Breast Cancer Study Group, The South-East Sweden Breast Cancer Group & the Gruppo Interdisciplinare Valutazione Interventi in Oncologica (G.I.V.I.O.). *Proc of ASCO*, vol. 18, 67a (abstr. 251).
Stewart HJ and Everington D (1995) Ovarian ablation versus CMF chemotherapy as adjuvant therapy for breast cancer. In Jonat W, Kaufmann M, Munk K (eds): Hormone-Dependent Tumors. Basic Research and Clinical Studies. Vol 50, pp 176–183 *Contrib Oncol.* Basel, Karger
Tamassia V, Battaglia A, Gangina F, Isetta AM, Sacchetti G, Cavalli F, Goldhirsch A, Brunner K, Bernardo G and Robustelli della Cuna G (1982) Pharmacokinetic approach to the selection of dose schedules for medroxyprogesterone-acetate in clinical oncology. *Cancer Chemother Pharmacol* **8**: 151–156
Tchekmedyian NS, Tait N and Aisner J (1986) High-dose megestrol acetate in the treatment of postmenopausal women with advanced breast cancer. *Semin Oncol* **13**: 20–25
Van Veelen H, Willemse PH, Sleijfer DT, Sluiter WJ and Doorenbos H (1985) Endocrine effects of medroxyprogesterone acetate: relation between plasma level and suppression of adrenal steroids in patients with breast cancer. *Cancer Treat Rep* **69**: 979–983
Van Veelen H, Willemse PHB, Tabies T, Sleijfer DT, Sluiter WJ and Doorenbos J (1986) Oral high-dose medroxyprogesterone acetate versus tamoxifene. A randomized cross-over trial in postmenopausal patients with advanced breast cancer. *Cancer* **58**: 7–13
Wils JA (1988) Myeloprotective effect of high dose medroxyprogesterone acetate (MPA). *Chemioterapia* **7**(1): 60–62

© 2001 Cancer Research Campaign
